# Interaction between CRHR1 and BDNF Genes Increases the Risk of Recurrent Major Depressive Disorder in Chinese Population

**DOI:** 10.1371/journal.pone.0028733

**Published:** 2011-12-14

**Authors:** Zheman Xiao, Wanhong Liu, Kai Gao, Qirong Wan, Can Yang, Huiling Wang, Xiaoping Wang, Gaohua Wang, Zhongchun Liu

**Affiliations:** 1 Institute of Neuropsychiatry, Wuhan University, Wuhan, China; 2 School of Medicine, Wuhan University, Wuhan, China; 3 Department of Psychiatry, Renmin Hospital, Wuhan University, Wuhan, China; Rikagaku Kenkyūsho Brain Science Institute, Japan

## Abstract

**Background:**

An important etiological hypothesis about depression is stress has neurotoxic effects that damage the hippocampal cells. Corticotropin-releasing hormone (CRH) regulates brain-derived neurotrophic factor (BDNF) expression through influencing cAMP and Ca2+ signaling pathways during the course. The aim of this study is to examine the single and combined effects of CRH receptor 1 (CRHR1) and BDNF genes in recurrent major depressive disorder (MDD).

**Methodology/Principal Finding:**

The sample consists of 181 patients with recurrent MDD and 186 healthy controls. Whether genetic variations interaction between CRHR1 and BDNF genes might be associated with increased susceptibility to recurrent MDD was studied by using a gene-based association analysis of single-nucleotide polymorphisms (SNPs). CRHR1 gene (rs1876828, rs242939 and rs242941) and BDNF gene (rs6265) were identified in the samples of patients diagnosed with recurrent MDD and matched controls. Allelic association between CRHR1 rs242939 and recurrent MDD was found in our sample (allelic: p = 0.018, genotypic: p = 0.022) with an Odds Ratio 0.454 (95% CI 0.266–0.775). A global test of these four haplotypes showed a significant difference between recurrent MDD group and control group (chi-2 = 13.117, df = 3, P = 0.016. Furthermore, BDNF and CRHR1 interactions were found in the significant 2-locus, gene–gene interaction models (p = 0.05) using a generalized multifactor dimensionality reduction (GMDR) method.

**Conclusion:**

Our results suggest that an interaction between CRHR1 and BDNF genes constitutes susceptibility to recurrent MDD.

## Introduction

Major depressive disorder (MDD) is frequently characterized by periodic depressed mood and the loss of interest, often with thoughts of death. Severe forms of depression affect 2–5% of the population worldwide, and up to 20% suffer from milder forms of the disease, and depression is also associated with high rates of relapse, recurrent, disability, and death [1]. Despite the high morbidity and mortality associated with MDD, the etiology and pathophysiology of MDD have not been precisely defined. Family, twin, and adoption studies provide strong evidence for an important genetic component [2]. To uncover the genetic mechanisms underlying susceptibility to depression and related traits may also prove a successful way to understand better the etiological features of MDD [3].

Stress response and neurotoxic effects are important etiological hypotheses about depression. Neurotoxins (possibly related to excessive corticotrophin activity and/or to the inflammatory effects of cytokines) damage or kill hippocampal cells, leading to many depressive symptoms. A deficient function of neuroprotective peptides, for example, brain-derived neurotrophic factor (BDNF), which reduces serum BDNF in MDD [4]. Hypothalamic-pituitary-adrenal (HPA) axis dysregulation and reduced neuroplasticity in depression are consistent with the assumption that BDNF is a stress-responsive intercellular messenger modifying HPA axis activity [5]. As a major mediator of the stress response in the central nervous system,corticotropin releasing hormone (CRH) affects other central processes, such as learning and memory, synaptic plasticity, and neuroprotection [6]. Abnormal CRH neurotransmission and receptor signal transduction has been proposed to be a critical mechanism for stress pathophysiology that leads to major depression [7]. Bayatti et al considered that CRH regulates BDNF expression through influencing cAMP and Ca2+ signaling pathways [8].

Based on different neuroanatomical expression patterns, there are two primary receptors subtypes in the central nervous system CRHR1 and CRHR2 [9]. CRH has a higher affinity for CRHR1 than for CRHR2, and in the brain, CRHR1 is expressed at high levels in the hippocampus, cortex and cerebellum [10]. CRH binding to CRHR1 typically activates adenylate cyclase (AC), which leads to increased intracellular concentrations of cAMP and activation of protein kinase A. One putative target is the BDNF, whose expression is controlled by cAMP-elevating agents in neurons [11]. In addition to its role as a classical target-derived growth factor during neuronal development, BDNF is an essential autocrine factor, released and acting locally after neuronal depolarization [12].

As CRHR1 may play a significant role in the etiology and treatment of depression, it is suggested that CRHR1 is a relevant candidate gene for MDD. In Mexican-Americans population, a significant association has been reported between CRHR1 and a greater response to selective serotonin reuptake inhibitors (SSRI) treatment in highly anxious MDD patients [13], but the distribution of the MDD was similar to the healthy controls. We have already reported that rs242939 AG carriers of CRHR1 have a greater risk of MDD in Han Chinese population, which showed marked geographical and ethnic variability of MDD [14], and that CRHR1 gene is likely to be involved in the antidepressant response in MDD [15]. Ressler et al reported a gene by gene-by-environment (G×G×E) interaction between the CRHR1, the 5-HTTLPR polymorphisms, and childhood abuse on depressive symptoms [16].

The BDNF gene rs6265 (G196A) polymorphism is functionally relevant [17]. The expression of BDNF is modified by antidepressant treatment [18]. Most association studies of BDNF polymorphism and depression related traits have yielded negative results. However, several lines of evidence suggest that lower active A allele of BDNF rs6265 polymorphism is associated with the features associated with the risk of depression [19].

Addressing gene-gene interactions is crucial to characterizing a trait involving complex disease-related mechanisms, particularly when each involved feature only demonstrates a minor marginal effect [20]. Gene-gene interactions have been hypothesized to contribute to the etiology of depression [21]. Considering the higher heritability of recurrent depression than single episode depression, analyzing these two groups separately was much more important [22]. On the basis of a large number of studies, we undertook a case-control study to examine the combined effects of CRHR1 and BDNF genes on recurrent MDD in Chinese Han people.

## Results

In our total sample size (n = 367), power analysis for case-control samples was carried out by G* Power program, the sample size had a post-hoc power of 0.99 to detect an effect size of 0.5 (moderate) at the 0.05 significance level (2-tailed). Genotype distributions for the four SNPs were in Hardy–Weinberg equilibrium. The results from single marker analysis of these four SNPs are presented in Table 1. An allelic association between CRHR1 rs242939 and recurrent MDD was found in our sample (allelic: p = 0.018, genotypic: p = 0.022) with an Odds Ratio of 0.454 (95% CI 0.266–0.775), which is reflected by a significant increase of the G-allele of 242939 in the recurrent MDD group than the control group. CRHR1 (rs1876828, rs242941) and BDNF (rs6265) alleles were found no association with the risk of recurrent MDD in present sample (p = 0.1952, 0.0822, 0.4078 respectively).

**Table 1:** Allele and genotype distribution of CRHR1 and BDNF polymorphisms of recurrent MDD patients and controls.

Gene	SNP ID^a^	Position	Genotype			P	Allele		P	Odds Ratio
										(95%CI)
			AA	AG	GG		A	G		
BDND	rs6265	chr11:27679916 MDD	39	87	55	0.4301	165	197	0.4078	1.134
		CON	37	84	65		158	214		(0.847–1.519)
CRHR1	rs1876828	chr17:43911525 MDD	149	30	2	0.3126	328	34	0.1952	1.395
		CON	143	39	4		325	47		(0.874–2.226)
	rs242939	chr17:43895579 MDD	139	40	2	**0.022** b	318	44	**0.018** b	0.454
		CON	166	18	2		350	22		(0.266–0.775)

			GG	GT	TT		G	T		
	rs242941	chr17:43892520 MDD	124	47	10	0.126	298	64	0.0822	1.421
		CON	115	58	13		285	87		(0.990–2.040)

a SNP, single nucleotide polymorphism.

b Bold numerals p-values after Bonferroni correction.

Haplotype frequencies in the MDD group and in the control group were estimated using the EM algorithm embedded. Four common haplotypes (G-A-G, G-A-T, A-A-T, G-G-T respectively the SNPs of rs1876828, rs242939, rs242941), were found to present in the sample. Using chi-2 test, a global test of these four haplotypes showed a significant different distribution between the recurrent MDD group and the control group (chi-2 = 13.104, df = 3, P = 0.016) (Table 2).

**Table 2:** Haplotype analysis of recurrent MDD patients and controls.

Haplotype	CON (%)^a,b^	MDD (%)^a,b^	chi-2	df	P -value
GAG	0.66	0.62	1.063	1	0.151
GAT	0.11	0.11	0.001	1	0.542
AAG	0.13	0.09	2.423	1	0.06
GGT	0.08	0.16	10.509	1	**0.003** c
Total			13.104	3	**0.016** c

a Haplotype frequencies (%) estimation using the EM algorithm from Arlequin.

b All haplotypes estimated to occur with a frequency of at least 1% in MD patients or controls are represented.

c Bold numerals p-values after Bonferroni correction.

To assess further combined effects of these genetic variants on recurrent MDD risk by using GMDR analysis. The GMDR software provides a number of output parameters, including CV consistency, the testing balanced accuracy, and empirical p values, to assess each selected interaction. The CV consistency score is a measure of the degree of consistency with which the selected interaction is identified as the best model among all possibilities considered. Furthermore, the testing balanced accuracy is a measure of the degree to which the interaction accurately predicts case–control status with scores between 0.50 (indicating that the model predicts no better than chance) and 1.00 (indicating perfect prediction). The interaction between rs6265 of BDNF and rs242939 CRHR1 had a CV consistency of 10 and p value of 0.05 after Bonferroni correction, which was considered as the best of two factors. The interaction between BDNF (rs6265) and CRHR1 (rs1876828, rs242939, rs242941) had a CV consistency of 10 and p value of 0.021, which was considered as the best of four factors, indicating a potential gene–gene interaction between BDNF and CRHR1. However, after Bonferroni correction, the 4-locus model no longer significant. Overall, the 2-locus model had the highest level of testing accuracy (59.16%) and showed good CV consistency (10/10) (Table 3). Therefore, we chose the 2-locus model as the best GMDR model. Furthermore, the significant interactions among the above 2-locus models were confirmed by logistic regression models (p = 0.013).

**Table 3:** Best gene–gene interactions models identified by the generalized multifactor dimensionality reduction (GMDR) method.

Locus number	Best combination	Cross-validation consistency	Testing accuracy (%)	P-value
2	rs6265, rs242939	10/10	59.16	**0.05** a
4	rs6265,rs1876828, rs242939, rs242941	10/10	57.52	**0.08** a

a Bold numerals p-values after Bonferroni correction.

## Discussion

In the present study, we evaluated the individual and the joint effects of four polymorphisms in two candidate genes on recurrent MDD in a Chinese population. The results suggested that the CRHR1 gene not only has a major effect, but also a combined effect with the BDNF locus on recurrent MDD. Our findings might provide further evidence that the CRHR/BDNF pathway play an important role in the etiology of recurrent MDD.

In the single-locus evaluation, it was found that the G-allele is more frequent in the recurrent MDD group than in the control group, which showed a statistically significant association of SNP rs242939 (P = 0.018) with recurrent MDD. By evaluating haplotype, rather than single-locus tests of association, the loss of information attributable to biallelic rather than multiallelic loci can be compensated, possibly resulting in an increased informativity. Haplotype analysis revealed that a haplotype of CRHR1had a highly significant positive association with recurrent MDD, a haplotype with alleles G-G-T respectively the SNPs of rs1876828, rs242939, rs242941, was significantly over represented in the recurrent MDD group than in the control group (chi-2 = 10.059, df = 1, P = 0.016), which suggested that carrying this haplotype might increase the probability of developing recurrent MDD in Han Chinese population.

A large amount of literature has focused on studying the genetic association between the BDNF gene and MDD. However, genetic association studies of the BDNF rs6265 on MDD have produced inconsistent results. BDNF polymorphism is associated with increased risk of depression in some studies [23], [24], while some studies found no association [25], [26], [27]. In the current study, using analysis of single loci, we did not find that BDNF rs6265 associated with recurrent MDD in Han Chinese population. There may be several interpretations for the discrepancy. Ethnic differences in polymorphism frequencies might contribute to inconsistent results in genetic association studies. Furthermore, MDD is a complex disease that is thought to be caused by multiple genetic factors each of small effect. Gene–gene interactions are likely to contribute to the pathophysiology of illness.

Using GMDR analyses, we further inferred the epistatic effects between CRHR1 and BDNF genes in recurrent MDD. We found a significant 2-locus (BDNF rs6265 and CRHR1 rs242939) gene–gene interaction models, which interactions confer an increased risk for recurrent MDD. Behavioral evidence supports that the CRHR1/BDNF system is involved in at least certain forms, such as learning and memory, synaptic plasticity [6]. The finding of CRHR1/BDNF interactions provides a better understanding of the genetic mechanisms of recurrent MDD.

Although evidence of a statistical interaction as we report here does not necessarily map directly onto a biological interaction, true gene-gene interactions nonetheless must have a biological basis [28]. Protein encoded by the CRHR1 and BDNF genes work together to result in lower BDNF in hippocampus. In depressed patients, a reduced volume of hippocampus has been reported, and this reduction in volume has been reversed by antidepressants [19]. There is also large number of studies suggesting a major role of BDNF in depression. BDNF rs6265 polymorphism is linked to substantial reduction in the volume of hippocampus, a brain structure repeatedly associated with the treatment response and neurogenesis in depression [29]. Our results further contribute to the hypothesized association between HPA axis dysregulation and reduced neuroplasticity in depression and are consistent with the assumption that BDNF is a stress-responsive intercellular messenger modifying HPA axis activity.

One limitation of this study is that the positive finding may be a result of type I error, but we addressed this limitation by using the Bonferroni correction. Second, our sample size is relatively small, however, our study had a power of 0.99 to detect an effect size of 0.5 (moderate) at the 0.05 significance level (2-tailed). Although the present study is preliminary, we suggested the interactional association of BDNF and CRHR1 in recurrent MDD should be replicated in large samples and in the other ethnic populations. Further work is required to investigate gene-gene interaction between other genes to progressively elucidate the pathophysiology involved in MDD.

## Materials and Methods

### Subjects

The patient sample consisted of 181 unrelated Chinese MDD individuals (male/female: 72/109; mean age: 33.68±10.07 years), who were outpatients and inpatients from the Psychiatric Department of the Renmin Hospital of Wuhan University. All patients had at least two well-defined episodes of MDD (average number: 3.6) as defined by DSM-IV [30]. Patients were interviewed by trained psychiatrists using the Structured Clinical Interview for DSM-IV disorders (SCID-I). Severity of depression was assessed using the 21-item Hamilton Rating Scale for Depression (HAMD-21) and the Clinical Global Impression Scale (CGI) [31], [32]. Only subjects with a minimum score of 18 on the HAMD-21 were selected. Patients with severe organic disorders or who showed comorbidity for other psychiatric disturbances (eg substance/alcohol dependence, personality disorders, anxiety disorders and others) were excluded. There were no significant differences concerning all other investigated variables (age, clinical variables such as CGI and HAMD-21 scores) in males and females.

A total of 186 (male/female: 71/115; mean age: 32.94±11.32 years) healthy controls whose age, gender, and ethnically matched subjects were selected from the general population (Table 4). All patients and controls were Han people who came from the same geographical region in China. The Medical Ethics Committees of the Renmin Hospital of Wuhan University approved the research project. Patients were included in the study after they gave written informed consent.

**Table 4:** Population characteristics of recurrent MDD patients and controls.

	Recurrent MDD	Controls
N	181	186
Age (mean±S.D.)	33.68±10.07	32.94±11.32
Gender (males/females)	72/109	71/115
Age of onset(males/females)	29.34±9.63	
Average onset times	3.6	
HAMD score	29.11±5.89	

### Genotyping

Genomic DNA was extracted from EDTA-anticoagulated venous blood samples which were carried out as described by Miller et al [33]. In this study, four single-nucleotide polymorphisms (SNPs) were assayed corresponding to the following dbSNP identifiers: rs1876828, rs242939 and rs242941 (CRHR1 gene), rs6265 (BDNF gene). SNPs were genotyped with TaqMan technology (Assay-by-Design) on an ABI 7900 system (Applied Biosystems). All MGB TaqMan probes and PCR primers were designed by Applied Biosystems (Foster City, CA, USA). The standard PCR reaction was carried out using TaqManR Universal PCR Master Mix reagent kit in a 5 µl volume. Fluorescence data files from each plate were analyzed by using automated software (SDS 2.1; Applied Biosystems). All laboratory procedures were carried out blind to case–control status.

### Statistical analysis

The GENEPOP program was used to compare the overall allele and genotype distributions for each SNP in MDD patients and controls, and to test Hardy–Weinberg equilibrium [34]. Haplotypes frequencies in recurrent MD patients and controls were estimated using the EM algorithm embedded in the program Arlequin [35]. A total of 10,000 permutation tests were performed in each analysis. Bonferroni correction was used for multiple testing, using the total number of SNPs as correction factor. The GMDR analysis was used to assess gene–gene interactions [36]. We reduced the n-dimensional space formed by a given set of SNPs to a single dimension to analyze *n*-way interactions. We calculated score-based statistics using maximum-likelihood estimates to classify multifactor cells into two different groups (either high risk or low risk). This was performed for all possible combinations of SNPs, and the combination with the lowest misclassification error was selected. Moreover, we tested all possible 2-locus to 4-locus interactions using 10-fold cross-validation (CV) in an exhaustive search, which considers all possible SNP combinations. In addition, we performed logistic regression models to confirm the results from GMDR analyses. Power analysis for case-control samples was carried out by G*Power program (alpha has been set at 0.05).
